# Automated extraction of functional biomarkers of verbal and ambulatory ability from multi-institutional clinical notes using large language models

**DOI:** 10.1186/s11689-025-09612-w

**Published:** 2025-04-30

**Authors:** Levi Kaster, Ethan Hillis, Inez Y. Oh, Bhooma R. Aravamuthan, Virginia C. Lanzotti, Casey R. Vickstrom, Brain Gene Registry Consortium, Inez Y. Oh, Virginia C. Lanzotti, M Wasserstein, M Chopra, M Sahin, M Wangler, B Schultz, K Izumi, S Bergner, A Gropman, C Smith-Hicks, L Abbeduto, H Hazlett, D Doherty, K German, L DaWalt, J Neul, J Constantino, D Baldridge, S Srivastava, S Molholm, S Walkley, E Storch, R Samaco, J Cohen, S Shankar, J Piven, S Mahida, A Sveden, K Dies, ER Riggs, JM Savatt, B Minor, Christina A. Gurnett, Philip R. O. Payne, Aditi Gupta, Christina A. Gurnett, Philip R. O. Payne, Aditi Gupta

**Affiliations:** 1 Institute for Informatics, Data Science and Biostatistics, Washington University School of Medicine in St. Louis, St. Louis, MO USA; 2 Department of Neurology, Washington University School of Medicine in St. Louis, St. Louis, MO USA; 3 Department of Psychiatry, Washington University School of Medicine in St. Louis, St. Louis, MO USA

**Keywords:** Functional biomarkers, Neurodevelopmental disorders, Electronic health records, Large language models

## Abstract

**Background:**

Functional biomarkers in neurodevelopmental disorders, such as verbal and ambulatory abilities, are essential for clinical care and research activities. Treatment planning, intervention monitoring, and identifying comorbid conditions in individuals with intellectual and developmental disabilities (IDDs) rely on standardized assessments of these abilities. However, traditional assessments impose a burden on patients and providers, often leading to longitudinal inconsistencies and inequities due to evolving guidelines and associated time–cost. Therefore, this study aimed to develop an automated approach to classify verbal and ambulatory abilities from EHR data of IDD and cerebral palsy (CP) patients. Application of large language models (LLMs) to clinical notes, which are rich in longitudinal data, may provide a low-burden pipeline for extracting functional biomarkers efficiently and accurately.

**Methods:**

Data from the multi-institutional National Brain Gene Registry (BGR) and a CP clinic cohort were utilized, comprising 3,245 notes from 125 individuals and 5,462 clinical notes from 260 individuals, respectively. Employing three LLMs—GPT-3.5 Turbo, GPT-4 Turbo, and GPT-4 Omni—we provided the models with a clinical note and utilized a detailed conversational format to prompt the models to answer: "Does the individual use any words?" and "Can the individual walk without aid?" These responses were evaluated against ground-truth abilities, which were established using neurobehavioral assessments collected for each dataset.

**Results:**

LLM pipelines demonstrated high accuracy (weighted-F1 scores > .90) in predicting ambulatory ability for both cohorts, likely due to the consistent use of Gross Motor Functional Classification System (GMFCS) as a consistent ground-truth standard. However, verbal ability predictions were more accurate in the BGR cohort, likely due to higher adherence between the prompt and ground-truth assessment questions. While LLMs can be computationally expensive, analysis of our protocol affirmed the cost effectiveness when applied to select notes from the EHR.

**Conclusions:**

LLMs are effective at extracting functional biomarkers from EHR data and broadly generalizable across variable note-taking practices and institutions. Individual verbal and ambulatory ability were accurately extracted, supporting the method's ability to streamline workflows by offering automated, efficient data extraction for patient care and research. Future studies are needed to extend this methodology to additional populations and to demonstrate more granular functional data classification.

**Supplementary Information:**

The online version contains supplementary material available at 10.1186/s11689-025-09612-w.

## Background

Verbal and ambulatory ability are functional and clinically meaningful biomarkers of intellectual and developmental disabilities (IDDs) that meet the National Institutes of Health Biomarkers Definitions Working Group definition as characteristics that are “objectively measured and evaluated as an indicator of normal biological processes, or pharmacological responses to a therapeutic intervention” [1]. Treatment planning, intervention monitoring, and identification of comorbid conditions in individuals with IDDs, as well as research to understand the underlying pathology of these conditions, currently relies on assessments of these functional ability biomarkers. However, standardized assessments, which may consist of either short user-defined surveys or well validated instruments or screening tools, are typically burdensome and time-consuming as they require dedicated effort from providers, specialists, affected individuals, and their caregivers for implementation, completion and storage. Furthermore, IDD phenotypes change over time, but standard assessments often provide only a one-time snapshot of an individual’s abilities and are challenging to accrue over time, limiting longitudinal clinical and research characterization of these phenotypes. In addition, standardized assessments may suffer from inconsistencies, including missing data, evolving guidelines/protocols, and patient/caregiver and assessor subjectivity, resulting in a lack of reproducibility. On the other hand, the electronic health record (EHR) constitutes a rich source of longitudinal real-world clinical data accrued obligately during routine healthcare encounters, including demographics, diagnoses, medications, procedures, laboratory results, vitals, and clinical notes, making the EHR a powerful resource for predicting health outcomes [2–4]. Hence, we propose an automated natural language processing (NLP) based pipeline to extract longitudinal functional phenotypes from EHR to augment and fill gaps in the information collected through formal assessments or surveys.

Verbal and ambulatory abilities are not often documented in structured tables within the EHR [5], despite recommendations by the US Department of Health and Human Services to document functional disabilities [6–9]. While deficient in structured data pertaining to functional ability, the EHR nevertheless possesses a wealth of unstructured and non-standardized information that may be helpful for identifying and characterizing disabilities. A study from 2011 examining the feasibility of manual abstraction of medical records to classify gross motor function of children with cerebral palsy (CP) records found that 90% of the study cohort had gross motor skill descriptions in their medical records that were adequate for Gross Motor Function Classification System (GMFCS) classification, with 75% agreement between two qualified clinician raters, suggesting that even without structured documentation, the EHR remains a valuable source of this information pertaining to functional ability [10].

Due to the diverse terminology and structures of clinical notes, the identification of verbal and ambulatory ability within notes is not trivial, and NLP techniques for clinical entity extraction are necessary to automatically derive this information from notes [2–4, 11]. Rule-based natural language processing methods have been widely utilized for clinical entity extraction because these methods identify exact pre-specified phrases found within the text, resulting in highly effective extraction [11–13]. However, identifying relevant phrases can be extremely time consuming, particularly when applying the same method across varying note authors, types and templates. Recently, Large Language Models (LLMs) have emerged as an alternative clinical entity extraction technique, with demonstrated success at information extraction from clinical texts [14, 15]. LLMs are pre-trained transformer-based artificial intelligence (AI) language models that have been utilized for a variety of NLP tasks [16, 17], and can be used out of the box without fine-tuning or intensive manual rule-development. Thus, we hypothesize that the identification of functional abilities from the EHR can be automated using state-of-the-art artificial intelligence methods, which may reduce patient/caregiver/provider burden and be an efficient tool to collect clinically important functional biomarkers for trending outcomes in natural history studies, assessing therapeutic responses, and identification of patients with functional disabilities who may benefit from targeted management strategies.

In this study, we leveraged LLMs to develop an automated and generalized pipeline to determine verbal and ambulatory ability using clinical notes from the EHR. We evaluated the performance of our pipeline in two independent cohorts: the national Brain Gene Registry (BGR), which includes multi-institutional participants with rare neurogenetic disorders, and a single institution (St. Louis Children’s Hospital Cerebral Palsy Center) cohort of individuals with CP, many of whom have functional limitations in verbal and motor abilities [18]. By implementing this pipeline on EHR data collected across twelve academic medical centers in the BGR and across two different clinical cohorts, we assess both the generalizability of the pipeline and its efficacy for predicting clinically meaningful functional biomarkers from EHR data. The goal of this work is to create a clinical extraction pipeline that can be generalized for extracting and analyzing other clinical phenotypes in IDDs more broadly.

## Methods

### Step 1: Data collection and preprocessing

#### National Brain Gene Registry (BGR) dataset

Data from the National BGR were obtained with the approval of the Washington University Institutional Review Board (# 202,010,013). The BGR aggregates clinically informative data from participants with rare genetic neurodevelopmental disorders, recruited across twelve academic medical centers with the goal of accelerating the curation of gene-disease associations [19, 20]. The repository includes genotypic and phenotypic data collected from a variety of sources, including patient EHR data, a Rapid Neurobehavioral Assessment Protocol (RNAP) that is comprised of a gold-standard battery of remotely delivered neurobehavioral assessments, spanning a variety of domains (including verbal and motor function)(Table 1) [20], variant-level genomic data obtained from GenomeConnect [21, 22], and additional relevant records such as previous neuropsychological reports, and photographs. As of May 8, 2024, the national BGR contained data from 564 participants recruited across 12 Eunice Kennedy Shriver Intellectual and Developmental Disability Research Centers (IDDRCs) [23].

**Table 1:** Assessments included in the rapid neurobehavioral assessment protocol

Rapid Neurobehavioral Assessment Protocol				
Domains Assessed	Measure	Assessment Type	Age	Time
Vision, Hearing and Verbal Ability	Telehealth Visit Guide	Parent Interview	all	5–10 min
Cognitive Ability	Shipley- 2, Block Patterns	Direct Assessment	7–89 yrs	10 min
	Developmental Profile- 4, Cognitive Domain	Parent Questionnaire	0–21 yrs, 11 mos	5–10 min
Adaptive Functioning	Vineland- 3: Comprehensive Parent/Caregiver Form	Parent Questionnaire	0–2 yrs, 11 mos	13–19 min
	Vineland- 3: Domain-Level Parent/Caregiver Form	Parent Questionnaire	3 yrs and older	12–18 min
Motor/Sensory Function	Repetitive Behavior Scale-Revised	Parent Questionnaire	2 yrs to adult	15 min
	Sensory Experiences Questionnaire- 3	Parent Questionnaire	2 yrs—12 yrs	15–20 min
	Developmental Coordination Disorder Questionnaire	Parent Questionnaire	5 yrs—15 yrs	10–15 min
	Gross Motor Functioning Classification System	Parent Questionnaire	2 yrs—18 yrs	< 5 min
Autistic Features	Social Communication Questionnaire-Lifetime Version	Parent Questionnaire	> 4 yrs	< 10 min
	Modified Checklist for Autism in Toddlers-Revised	Parent Questionnaire	16 mos—30 mos	10 min
	Social Responsiveness Scale- 2	Parent Questionnaire	2.5 yrs—19 + yrs	15–20 min
	Childhood Autism Rating Scale- 2 Observer (High Functioning or Standard)	Direct Assessment	all	15–20 min
Psychiatric Symptoms	Vanderbilt ADHD	Parent Questionnaire	6 yrs—12 yrs	10 min
	Achenbach System of Empirically Based Assessment (ASEBA)	Parent Questionnaire	1.5 yrs—59 yrs	15–20 min
	Abberant Behavior Checklist-Community	Parent Questionnaire	5 yrs—adult	10–15 min
Physical Features	Dysmorphology Screen	Direct Assessment	all	10–15 min
	Photographs	Parent Completed	all	10–15 min
Neurological Symptoms	Seizure History	Parent Questionnaire	all	10–15 min
	Virtual Neurological Exam	Direct Assessment	All	10–15 min
	Edinburgh Handedness Inventory	Parent Questionnaire	4 yrs—adult	5 min

Table depicting information relevant to the neurobehavioral assessment measures included in the RNAP, such as the measure name, the domain assessed, how the assessment is performed, the age range of individuals the measure can be applied to, and the approximate time taken complete the assessment

For this study we extracted data from sources in the BGR:

- Clinical Notes from EHR: We focused on progress notes, corresponding note metadata (author name, encounter date, note type, etc.), and demographics (DOB, sex, etc.) originating from the electronic health record (EHR); and questionaries, surveys and other neurobehavioral assessments collected through the BGR’s RNAP. The clinical notes obtained from the BGR were authored between July 2002 to March 2024. In sum, 43,482 clinical notes were included in the initial data pull from the BGR, which we narrowed down to 19,546 notes after selecting only progress notes. We further limited the dataset to progress notes most relevant to recent verbal and ambulatory status based on these criteria: 1) notes authored by providers from 14 specialties determined by expert clinicians to be relevant according to note type and frequency, 2) notes of individuals who had ground-truth labels (i.e. RNAP data), 3) notes written when the individual was at least 3 years old, and 4) notes of individuals who have at least 5 notes. The 14 specialties whose notes were determined by expert clinicians to be relevant are listed in Supplementary Table 1. After applying these criteria, the final dataset consisted of 3,245 notes from 125 individuals.
- Neurobehavioral Assessments: Every participant in the BGR had their RNAP data collected between February 2021 and May 2024. The RNAP data was utilized as ground-truth labels to evaluate the performance of the NLP pipeline developed to extract functional phenotypes from EHR clinical notes. Table 1 summarizes all assessments included in the RNAP.

#### Cerebral palsy (CP) dataset

Data for the CP cohort were obtained with the approval of the Washington University Institutional Review Board (# 202,309,003), and two databases from the St Louis Children’s Hospital CP Center cohort were utilized:

- Research Database with Standardized Assessments: These data included St. Louis Children’s Hospital (SLCH) CP Center provider-populated GMFCS, Viking Speech Scale (VSS), and Communication Function Classification System (CFCS) classifications, which are well validated batteries for classifying self-initiated motor, speech, and communication abilities, respectively [24–29]. Since April 2023, providers in the St. Louis Children’s Hospital CP Center have routinely assessed and documented these on all patients, thus generating a high-quality ground-truth labels for evaluating our pipeline for extracting and mapping verbal and motor function data from EHR data to these scales.
- Clinical Notes from the EHR: Clinical notes authored between 9/22/2022—8/26/2024 were extracted from Washington University School of Medicine’s (WUSM’s) Research Data Core, a research repository of EHR data originating from the WUSM/Barnes Jewish Hospital/St. Louis Children’s Hospital Epic EHR system. In total, 134,177 clinical notes were included in the initial CP data pull, of which 19,551 were progress notes. We further reduced the dataset to only notes relevant to gross motor function classification based on these criteria: 1) notes from the same 14 specialties as the BGR dataset, 2) notes written within 1.5 years of when the ground-truth annotations were created for each patient, 3) notes containing at least 500 words, 4) notes of individuals who have at least 5 remaining notes. This resulted in a dataset of 5,462 notes. In the next step, from these notes we removed any references of the GMFCS and VSS score in order to mitigate bias and ensure that our pipeline predicts functional phenotypes without using the values from these instruments. Using string search, the following phrases and the 15 characters before and after were removed to prevent data leakage: ‘gmfcs’, ‘gross motor function’, ‘gross motor function cs’, ‘vss’, ‘viking speech’, and ‘cfcs’. The final dataset consisted of 5,462 notes from 260 individuals.

### Step 2: Targeted functional biomarkers for extraction

**Fig. 1:**
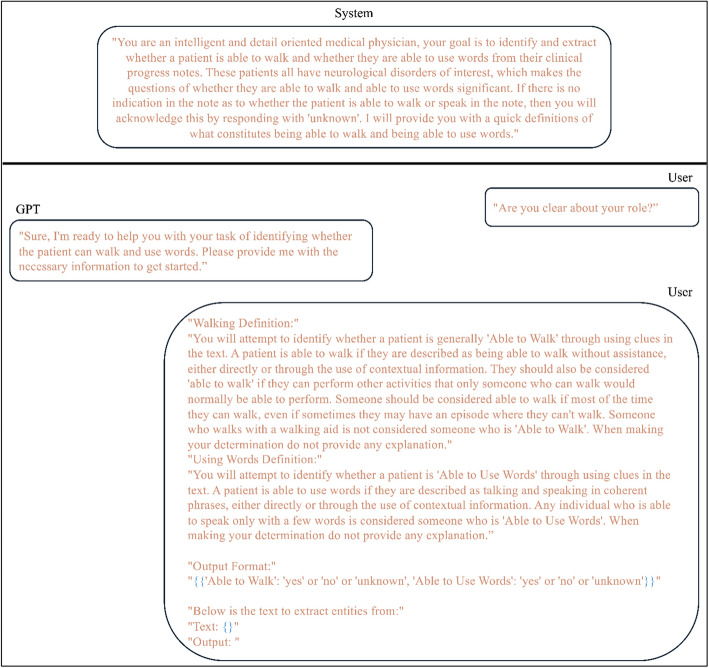
Prompt for large language model analysis. The generative pre-trained transformer (GPT) model was prompted in a conversational format in which GPT’s system prompt is first asserted. The system prompt steers the behavior of the model, allowing for it to be more adaptable to the task. The user (researcher) then asks if GPT understands its role, to which GPT confirms. Finally, the user provides detailed walking and using words definitions and extraction instructions with the desired output format. The clinical note is then included in the prompt at the placeholder symbol “{}”

We identified two functional biomarkers of interest which were the ability to use any number of words via motor speech (verbal ability) and ability to walk independently without any assistance or walking devices (ambulatory ability), for extraction from the clinical notes in our datasets. To extract these, we developed two questions to inform our prompt to the LLM, “Does the individual use any words?” and “Can the individual walk without aid?”. An initial prompt was developed to elicit an LLM response to these questions when provided a clinical note. By testing against a small subset of notes, iterative prompt engineering was performed to maximize performance. For example, the LLM originally mistakenly included those walking with assistance as those able to walk, so the clarifying text “someone who walks with a walking aid should not be considered ‘able to walk’” was added to our prompt. The final prompt was reviewed by clinicians and informaticians with NLP experience to ensure accuracy. The iteratively achieved final prompt consisted of 4 components: (1) a system prompt informing the model of its role to emulate a medical physician with the goal to extract verbal and ambulatory ability from a clinical note, (2) definitions of verbal and ambulatory ability, (3) an example of the output format, and (4) the clinical note text. This full prompt is shown in Fig. 1.

### Step 3: Identification of ground-truth functional biomarker status of individuals

#### Ground-truth for the Brain Gene Registry (BGR) dataset

The RNAP in the BGR includes questions directly relevant to verbal and ambulatory ability, allowing us to establish individual-level ground-truth verbal and ambulatory status without manually annotating clinical notes. Relevant surveys and assessments included the second edition of the Child Autism Rating Scale (CARS- 2) [30], the Gross Motor Function Classification System (GMFCS) [31], a telehealth screener, and the Modified Checklist for Autism in Toddlers, Revised (M-CHAT-R) [32, 33]. We mapped responses from these assessments to ground-truth labels for the two selected questions “Does the individual use any words?” and “Can the individual walk without aid?”, which are provided in Table 2. There were no conflicting mapped responses among participants who completed multiple assessments that map to a single identified question (i.e. CARS- 2 and Telehealth Screener).

**Table 2:** RNAP assessment responses mapped to identified questions

Assessment	Identified Question	Assessment Question	Response	Mapped Response
CARS- 2	*Does the individual use any words?*	Is the person you are rating using words?	Yes	Yes
			No	No
Telehealth Screener		Does [participant’s name] use single words?	Yes	Yes
			No	No
GMFCS	*Can the individual walk without aid?*	Motor function is classified on a 1–5 scale according to different criteria for different ages	Class: 1 Age: 3 to 4	Yes
			Class: 2,3,4,5 Age: 3 to 4	No
			Class: 1,2 Age: 4 to 18	Yes
			Class: 3,4,5 Age: 4 to18	No
M-CHAT-R		Does your child walk?	Yes	Yes
			No	No

This table lists the RNAP assessments utilized to determine the patients’ ground-truth verbal and ambulatory abilities. Columns include the name of the assessment, the specific verbal or ambulatory ability question that is addressed, the pertinent question from the assessment, the recorded response to the assessment question, and the mapped ground-truth patient verbal or ambulatory ability

#### Ground-truth for the Cerebral Palsy (CP) dataset

The ground-truth CP annotations are solely based on GMFCS, the Viking Speech Scale (VSS), and the Communication Function Classification System (CFCS). The mappings to the identified questions are provided in Table 3. The ambulatory status predictions were evaluated once against the GMFCS; however, the verbal status predictions were evaluated separately against both the VSS and CFCS.

**Table 3:** Cerebral palsy assessment responses mapped to identified questions

Assessment	Identified Question	Assessment Question	Response	Mapped Response
VSS	*Does the individual use any words?*	Motor impact on speech is classified on a 1–4 scale	Class: 1, 2, 3	Yes
			Class: 4	No
CFCS		Communication ability with familiar and unfamiliar people at levels 1 through 5	Class: 1, 2, 3, 4	Yes
			Class: 5	No
GMFCS	*Can the individual walk without aid?*	Motor function is classified at levels 1 through 5 using different criteria for different ages	Class: 1	Yes
			Class: 2,3,4,5 Age: 3 to 4	No
			Class: 1,2 Age: 4 to 18	Yes
			Class: 3,4,5 Age: 4 to18	No

This table lists the assessments utilized to determine the patients’ ground-truth verbal and ambulatory abilities for the Cerebral Palsy cohort. Columns include the name of the assessment, the specific verbal or ambulatory ability question that is addressed, the pertinent question from the assessment, the recorded response to the assessment question, and the mapped ground-truth patient verbal or ambulatory ability

### Step 4: Development of NLP/LLM pipeline to identify individual functional biomarker status

Three pipelines were built to prompt the *Generative Pre-trained Transformer (GPT) model* to classify patient verbal and ambulatory status at the note level, utilizing GPT- 3.5 Turbo version 0613 (GPT- 3.5), GPT- 4 Turbo model version 0125-preview (GPT- 4 t), or GPT- 4 Omni (GPT- 4o). Each pipeline employed HIPAA-compliant OpenAI endpoints of GPT accessed through Washington University’s Azure tenant, allowing clinical notes to be provided to the model without prior deidentification. Each clinical progress note was provided to the model, which was prompted to utilize only the information found in the note to answer: “Does the individual use any words?” and “Can the individual walk without aid?*”* Full unmodified progress notes were provided to the model when using GPT- 4 t and GPT- 4o, but the smaller context limit of GPT- 3.5 required truncated note versions (10,000 characters) to be provided when using that model, and the rest of the note was discarded. Two separate prompts were developed for each pipeline: a multi-class prompt (MCP) that allowed GPT to respond with “yes”, “no”, or “unknown” to both questions concurrently, and a binary-class prompt (BCP) that restricted GPT responses to “yes” or “no”. To develop these prompts, we selected a subset of 10 notes from the BGR representing unique specialties and applied a baseline prompt to these notes. Then we iteratively refined the prompts to ensure that note-level extraction matched the note content, resulting in the final prompts that we used. For each version of GPT, the verbal and ambulatory ability were extracted for three unique experiments consisting of different combinations of prompts and note sets, testing the effects of allowing GPT to respond with ‘unknown’ and evaluating whether notes written more closely in time to the creation of the ground-truth labels contain more relevant information. The three unique experiments are below:

1. MCP-All: Prompt GPT to perform multi-class extraction (“yes”, “no”, and “unknown”) on the entire set of identified clinical notes.
2. BCP-All: Prompt GPT to perform binary-class extraction (“yes” or “no”) on the entire set of identified clinical notes.
3. MCP- 1.5Y: Prompt GPT to perform multi-class extraction on only the clinical notes written within 1.5 years of the date the patient was enrolled in the respective registries.

Each of these experiments were evaluated on the BGR cohort data, but only the MCP- 1.5Y prompt and note set was evaluated on the CP cohort data. Binary class prediction was excluded from the CP cohort analysis because we found the multi-class prediction prompts led to better or matching performance in the BGR data, as seen in Table 5**.** Prediction on the entire CP cohort was not performed due to the larger note counts within the CP cohort compared to the BGR cohort, as well as cost constraints associated with a larger note cohort (see *Cost Analysis*).

#### Predict functional biomarker status per individual

For the BGR and CP datasets, we mapped the note-level predictions to a single individual-level prediction because the ground-truth labels were known for each individual. For each question and individual in the BGR, the mapped response was “yes” if the number of notes with a “yes” prediction was greater than the number of notes with a “no” prediction, and the mapped response was “no” otherwise. The content and contextual patterns of notes from the CP cohort differed from the BGR dataset, which necessitated a different methodology for mapping note-level ambulatory predictions to individual-level predictions. This was because many individuals in the CP cohort had weekly physical therapy notes with identical note history sections, which cause an influx of “no” ambulatory predictions for these individuals. Therefore, for individuals in the CP cohort, the mapped ambulatory prediction response was “yes” if there was at least one note with a “yes” prediction, and the mapped response was “no” otherwise. For verbal predictions, the same mapping methodology as BGR was used.

### Step 5: Evaluation of pipeline

**Fig. 2:**
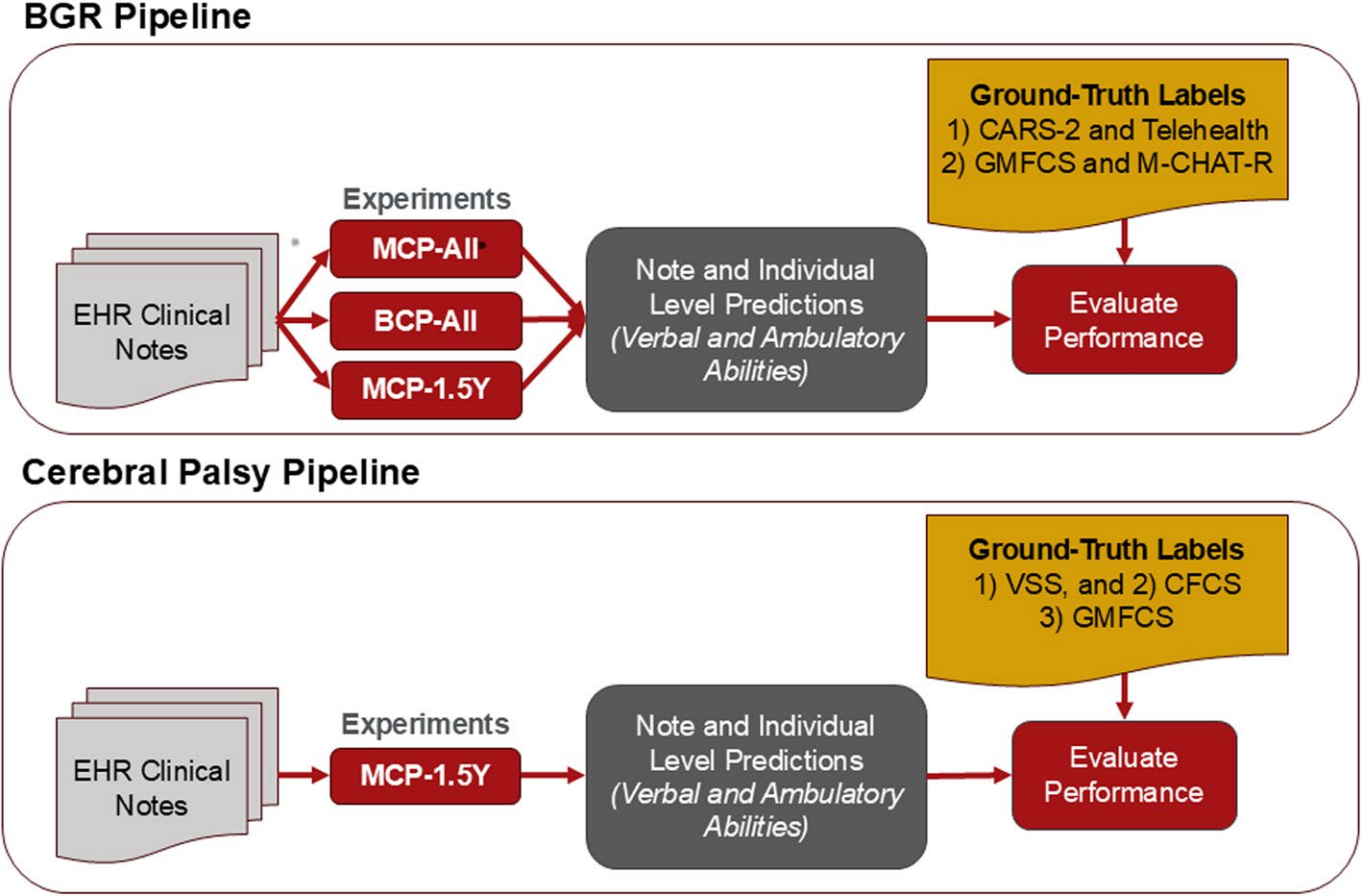
Illustration of GPT project workflow for both cohorts. The pipelines in the photo are repeated for all versions of GPT utilized: GPT- 3.5, GPT- 4 t, and GPT- 4o

The above analyses were performed on both the BGR and CP datasets except, as indicated earlier, only the MCP- 1.5Y experiment was performed for the CP dataset. The final functional biomarker status prediction for each individual in the BGR and CP datasets was evaluated against the RNAP-informed ground-truth labels or the CP Center-curated ground-truth labels, respectively, using metrics including average precision, average recall, weighted-average F1 score, and macro-averaged F1 scores. The weighted-average F1 score is calculated as $\left(F1-Score"Yes"Class\right)\ast\left(\frac{"\mathrm{Yes}"\;\mathrm{Annotations}}{Total\;Annotations}\right)+\left(F1\;Score-"No"Class\right)\;\ast\;(\frac{"No"Annotations}{Total\;Annotations})$, and was chosen to give more weight to classes with more data points, which we felt represented model performance accurately given the imbalance within the BGR dataset between ‘yes’ and ‘no’ annotations. This weighted score is calculated as Fig. 2 summarizes the NLP/LLM pipelines and evaluations applied to the clinical notes from the BGR and CP cohorts.

#### Cost analysis

Utilizing OpenAI’s GPT endpoint is a significant expense, as there is a per token cost associated with providing the note and prompt to GPT, as well as a per token cost to GPT’s output [34]. In the interest of transparency for those who may want to use GPT for similar tasks, we provide our anticipated and actual costs of Azure services associated with the project in Table 7. To calculate our expected costs, we first found the average number of characters in our prompt (which includes the provided clinical notes) and in GPT’s expected output. Next, we calculated the average token size of our inputs and outputs using OpenAI’s estimation of 4 characters per token, which we used to calculate the cost of each API call. Finally, we multiplied by the total number of notes to get the cost of generating a single round of predictions. For the BGR cohort there were two total rounds of predictions per GPT version and dataset, consisting of the MCP-All and BCP-All experiments. A separate analysis was not needed for MCP- 1.5Y, as the results from the multi-class predictions on all notes could be restricted to only notes within 1.5 years of enrollment. For the CP cohort, only one round of predictions was needed since a binary-class prediction experiment was not utilized.

The steps described above are represented in the *expected total cost* equation that we used below, which is the total cost for each model (GPT- 3.5, 4 or 4o) for the specific cohort. The results of the cost analysis are found in Table 6 in the results.

$$\begin{array}{c}Total\;Cost=P\cdot\left[N\cdot\left(\frac{\left\{Avg\;Input\;Chars\right\}}4\cdot\frac{\left\{Input\;Cost\right\}}{1000}+\frac{\left\{Output\;Cost\right\}}{1000}\cdot TpO\right)\right]\\N=\#\;of\;Notes\;\left|\mathit4=Chars\;per\;Token\right|\;1000=Convert\;to\;Cost\;per\;single\;token\\TpO=Expected\;Tokens\;Per\;Output\;\left|P=\#\;Prompts\;Used\;Per\;Model/Note\right|\end{array}$$

For the verbal and ambulatory status classification of notes from the BGR, the N was 3245, the P was 2 (MCP-All and BCP-All), and the TpO was 13. For the classification of notes from the CP cohort the N was 5,462, the P was 1 (MCP- 1.5Y), and the TpO was 13.

## Results

### Demographics

As shown in Table 4, the BGR cohort included 564 individuals, of which subsets of 104 and 105 met the inclusion criteria based on identified clinical progress notes and ground-truth RNAP information for patient ambulatory and verbal status, respectively. These subsets encompassed 125 unique individuals whose notes were provided to GPT. These individuals had an average age of 11.2 years, were a majority male (67.2%), and had a race breakdown of 4% Asian, 6.4% Black or African American, 73.6% White, 2.4% more than one race, and 13.6% other/unknown/not reported. Overall, the final dataset of individuals with ground-truth labels consisted of a greater percentage of individuals who are white or male than the broader BGR dataset, which is 54.1% male and 67.6% White. Supplementary Table 2 provides a more detailed breakdown of the BGR cohort demographics, compared according to the ground-truth labels.

**Table 4:** Demographics of BGR and CP cohorts

		BGR: All Individuals in Cohort	BGR: Individuals in GPT Dataset	CP: All Individuals in Cohort	CP: Individuals in GPT Dataset
N		564	125	633	260
Age (sd)		11.4 (9.8)	11.2 (6.1)	8.5 (5.1)	7.53 (4.9)
Sex	Female	259 (45.9%)	41 (32.8%)	292 (46.1%)	124 (47.7%)
	Male	305 (54.1%)	84 (67.2%)	337 (53.2%)	135 (51.9%)
	Unknown/Not Reported	0 (0.0%)	0 (0.00%)	4 (0.6%)	1 (0.4%)
Ethnicity	Hispanic or Latine	56 (9.9%)	8 (6.4%)	N/A	N/A
	Not Hispanic or Latine	406 (72.0%)	94 (75.2%)	N/A	N/A
	Unknown/Not Reported	102 (18.1%)	23 (18.4%)	N/A	N/A
Race	Asian	25 (4.4%)	5 (4.0%)	12 (1.9%)	7 (2.7%)
	Black or African American	27 (4.8%)	8 (6.4%)	113 (17.9%)	62 (23.9%)
	More Than One Race	23 (4.1%)	3 (2.4%)	31 (4.9%)	20 (7.7%)
	Other	19 (3.4%)	1 (0.8%)	12 (1.9%)	5 (1.9%)
	Unknown/Not Reported	88 (15.6%)	16 (12.8%)	24 (3.8%)	10 (3.9%)
	White	381 (67.6%)	92 (73.6%)	492 (77.7%)	192 (73.9%)

Demographics are provided for entire cohorts and just the subsection that the pipelines were applied to. Data include self-reported sex, ethnicity, race, and age

The CP cohort consisted of 633 individuals, all of whom had clinical notes and ground-truth GMFCS, VSS, and CFCS scale annotations mapped to binary output (Table 5). After the note inclusion criteria were applied, the cohort consisted of 260 individuals, with 125 able and 135 unable to walk without aid, as shown in Supplementary Table 3, which provides a more detailed demographic breakdown. These individuals had an average age of 7.5 years, were 51.9% male, and had a race breakdown of 2.7% Asian, 23.9% Black or African American, 73.9% White, 7.7% Mixed race, and 5.8% other/unknown.

**Table 5:** BGR dataset GPT performance

Extraction Task	Ground Truth Labels	Experi-ment	GPT 3.5			GPT- 4 t			GPT- 4o		
			Avg Precision	Avg Recall	Weighted Avg F1	Avg Precision	Avg Recall	Weighted Avg F1	Avg Precision	Avg Recall	Weighted Avg F1
Verbal Ability	RNAP (CARS- 2 or Telehealth Screener)	MCP-All	.80	.89	.88	.88	.84	.91	**.87**	**.88**	**.92**
		BCP- All	.78	.83	.87	**.84**	**.84**	**.91**	.82	.84	.89
		MCP- 1.5Y	.71	.76	.82	.80	.79	.86	**.82**	**.84**	**.89**
Ambulatory Ability	RNAP (GMFCS or M-CHAT-R	MCP-All	.60	.69	.75	**.89**	**.91**	**.95**	.75	.87	.89
		BCP- All	.52	.54	.45	**.67**	**.81**	**.81**	.63	.76	.74
		MCP- 1.5Y	.63	.74	.78	**.84**	**.86**	**.94**	.74	.83	.88

The precision, recall, and weighted-average F1 scores are reported across 3 GPT versions (GPT- 3.5, GPT- 4 t, ad GPT- 4o) and for verbal and ambulatory extraction tasks. Additionally, results are provided for each experiment, including the multi-class prediction experiment (MCP-All), the binary class prediction experiment (BCP-All), and the multi-class prediction experiment on notes written within 1.5 years of patient enrollment in the BGR (MCP- 1.5Y). The top scoring GPT model for each experiment in the table is bolded, and the top scoring experiment and GPT model combination for each extraction task is underlined

### GPT classification performance on BGR dataset

GPT was prompted to answer the questions ‘Can the individual walk without aid?’ and ‘Does the individual use any words?’ for all clinical notes in the three note/prompt sets, which included the multi-class classification prompt for all identified notes (MCP-All) and the multi-class classification prompt for notes written within 1.5 years of enrollment in the BGR (MCP- 1.5Y). These note level responses were mapped to individual-level predictions, which were then compared to the ground-truth labels as determined by the RNAP. Model performances are displayed in Table 5, representing ambulatory status and verbal ability classification respectively. The GPT model with the highest F1 scores for each experiment is highlighted in each table. The performances with macro-averaged F1 scores are available in Supplementary Table 4.

GPT models were successfully able to complete both tasks, with a maximum weighted F1 score of 0.95 being achieved for classifying ambulatory status, and a maximum score of 0.92 for identifying verbal ability. The highest performing GPT model was question dependent, with GPT- 4 t outperforming GPT- 4o at classifying ambulatory ability, whereas GPT- 4o generally outperformed GPT- 4 t at identifying ambulatory status. GPT- 3.5 was worst performing model for both tasks and across all experimental combinations, achieving a maximum performance of 0.78 for ambulatory classification and a higher maximum performance of 0.88 for verbal ability classification. Finally, we highlight that performance was slightly increased when the entire set of identified clinical notes was provided to models, as opposed to only notes written within one and a half years of BGR enrollment; however, this increase was not found to be significant utilizing the Wilcoxon signed-rank test.

### GPT classification performance on CP dataset

GPT was applied to the CP dataset and prompted to answer the questions ‘Can the individual walk without aid?’ (MCP- 1.5Y, GMFCS) and ‘Does the individual use any words?’ (MCP- 1.5Y, VSS) and (MCP- 1.5Y, CFCS) for notes written within 1.5 years of ground truth generation. The performance of GPT at determining verbal ability in this dataset was evaluated twice, first using the VSS as ground-truth labels, and second using CFCS. Model performances are displayed in Table 6, representing both verbal and ambulatory status. The results with macro-averaged F1 scores are available in Supplementary Table 5.

**Table 6:** CP dataset GPT performance

Extraction Task	Ground Truth Labels	Experi-ment	GPT 3.5			GPT- 4 t			GPT- 4o		
			Avg Precision	Avg Recall	Weighted Avg F1	Avg Precision	Avg Recall	Weighted Avg F1	Avg Precision	Avg Recall	Weighted Avg F1
Verbal Ability	VSS	MCP- 1.5Y	.69	.63	.51	.72	.71	.66	**.74**	**.73**	**.68**
	CFCS	MCP- 1.5Y	.69	.63	.52	.68	.67	.63	**.72**	**.71**	**.66**
Ambulatory Ability	GMFCS	MCP- 1.5Y	.73	.75	.69	.86	0.9	.88	**.88**	**.91**	**.90**

The precision, recall, and weighted-average F1 scores are reported across 3 GPT versions (GPT- 3.5, GPT- 4 t, ad GPT- 4o) and for verbal and ambulatory extraction tasks. All extractions were performed using multi-class prediction on notes written within 1.5 years of patient enrollment in the registry. For the verbal ability extraction, the extraction results utilizing ground truth labels from both the VSS and CFCS assessments were evaluated. The top scoring GPT model ground truth label set is bolded, and the top scoring label and GPT model combination for each extraction task is underlined

GPT models achieved a maximum weighted F1 of 0.90 for classifying ambulatory status, and a maximum weighted F1 score of 0.68 for classifying verbal status. GPT- 4o was the highest performing model, outperforming both GPT- 4 t and GPT- 3.5. GPT- 3.5 had the lowest performance with maximum weighted F1 scores of 0.69 and 0.52 for ambulatory and verbal status respectively.

### BGR vs CP GPT performance comparison

For ambulatory function prediction, performance on the 1.5Y note cohort between the BGR and CP cohorts was comparable. All GPT models for the CP cohort had higher recall, sensitivity, and macro averaged F1 scores, while the BGR cohort had higher weighted F1 scores. The CP models having higher macro averaged F1 and lower weighted F1 scores indicates that CP models had better general performance for both positive and negative ambulatory classes while the BGR models had better performance on the majority positive ambulatory class. This showcases the high degree of prompt generalizability for predicting ambulatory status.

However, for verbal status prediction, GPT performances on the CP cohort fell short of its performance on the BGR cohort performances. Across the three GPT models, for both VSS and CFCS labels, the verbal weighted F1 scores on the BGR cohort ranged from 0.17 to 0.31 greater than the weighted F1 scores on the CP cohort.

### Extraction performance across note types

**Fig. 3:**
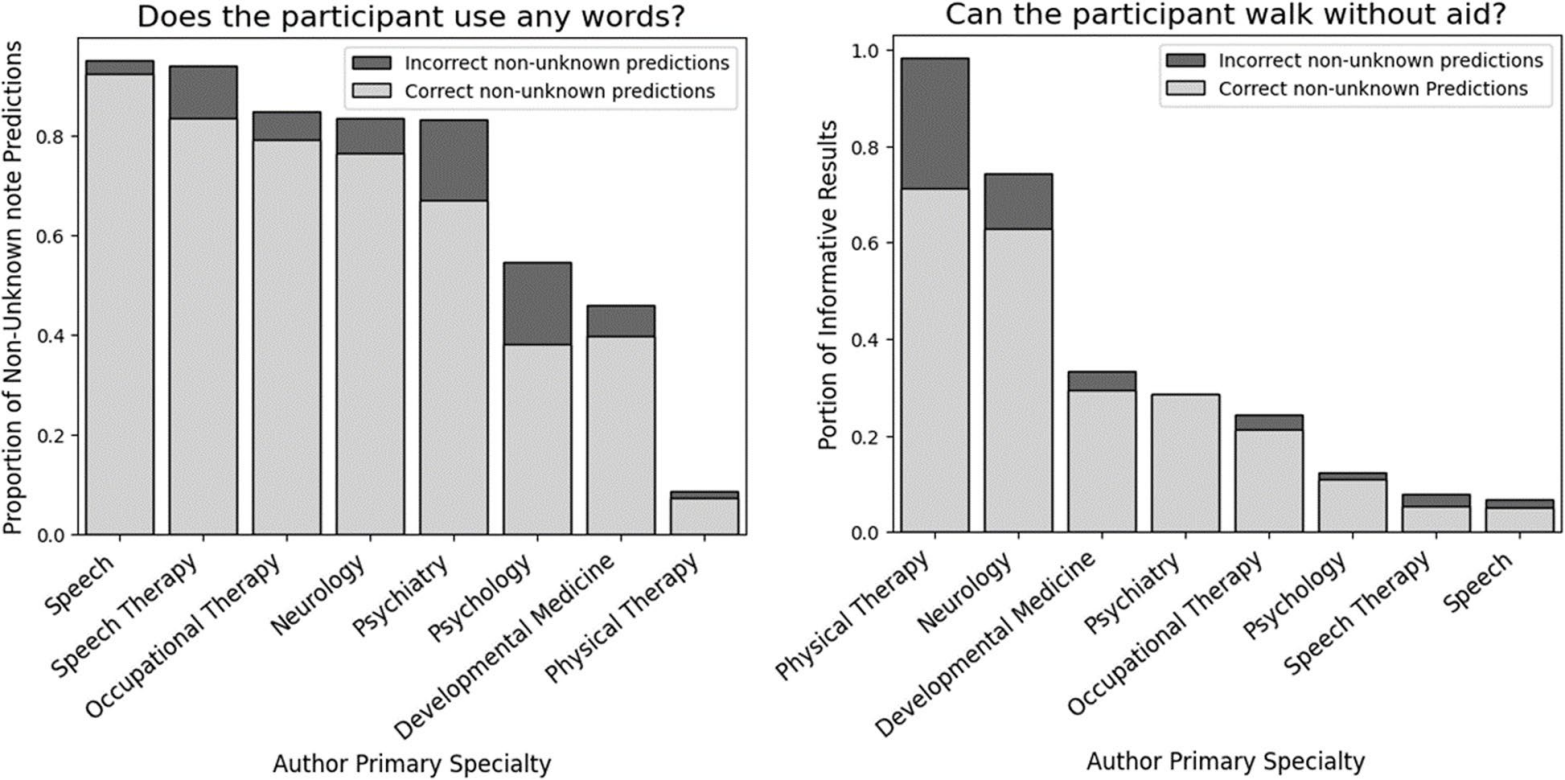
Proportion and correctness of non-unknown GPT note-level BGR predictions

To evaluate the most informative notes for the prediction of each functional biomarker, we compared the note-level GPT predictions for each note type to the individual ground-truth values. Figure 3 displays this analysis for the BGR cohort, demonstrating the correctness of each note type at extracting verbal and ambulatory status, and the proportion of the time that GPT predicts ‘unknown’. We found that for verbal status prediction, the speech therapy and speech notes output non-unknown (‘yes’ or ‘no) predictions at the highest prevalence, suggesting they are most informative for this extraction. For the ambulatory status extraction, the physical therapy notes led to non-unknown predictions at the highest prevalence. There was no obvious general relationship between the proportion of non-unknown predictions by note-type, and the correctness of these non-unknown predictions.

**Fig. 4:**
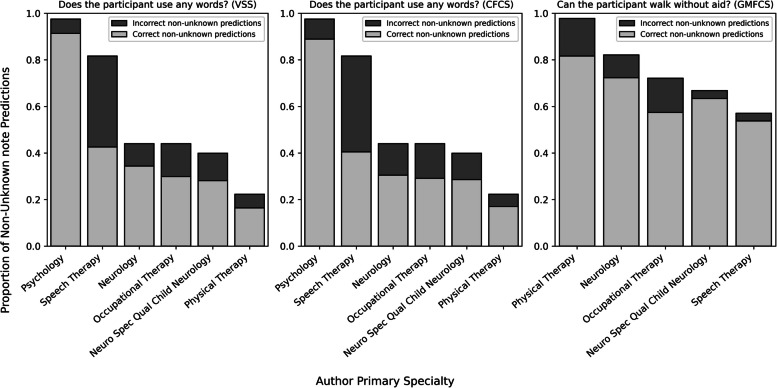
Proportion and correctness of non-unknown GPT note-level CP predictions

Similar findings are found in the CP cohort (Fig. 4). In this cohort, the note types leading to the lowest portion of unknown predictions for verbal ability extraction were psychology and speech therapy notes. As observed in the BGR cohort, the physical therapy notes were the most informative note type for ambulatory ability extraction. Though there was no relationship between proportion of non-unknown predictions and the correctness of predictions, the correctness of the predictions varied widely. For example, the verbal status predictions originating from the psychology notes were over 90% correct, whereas the non-unknown predictions from speech therapy notes were worse than random guessing.

For each note type, this figure displays the proportion of correct note-level predictions of the BGR cohort notes in which there was a non-unknown (i.e. “yes” or “no”) prediction. The full bar length shows the proportion of that note type that had non-unknown predictions. The dark grey bars represent the incorrect note-level predictions with light grey bars representing the correct note-level predictions. The note level GPT prediction was considered correct if the prediction matched the label for the corresponding individual. This figure was generated using the MCP- 1.5Y (notes within 1.5 years enrollment and multi-class classification) experiment and *GPT- 4o.* Out of the 14 relevant note types identified, only note types with a sample size of at least 50 notes of that type in our dataset are displayed in this figure.

For each note type, this figure displays the proportion of correct note-level predictions of the CP cohort notes in which there was a non-unknown (i.e. “yes” or “no”) prediction. The full bar length shows the proportion of that note type that had non-unknown predictions. The dark grey bars represent the incorrect note-level predictions with light grey bars representing the correct note-level predictions. The note level GPT prediction was considered correct if the prediction matched the label for the corresponding individual. This figure was generated using the MCP- 1.5Y (notes within 1.5 years enrollment and multi-class classification) experiment and GPT- 4o. Only note types with a sample size of at least 50 predictions are included in this figure.

### Cost analysis

We performed a cost estimate for each model and dataset combination using the equation described in the methods, showing that GPT- 4 t was expected to be the most expensive analysis, followed by GPT- 4o, and then GPT- 3.5 (Table 7).

**Table 7:** GPT cost analysis

Dataset	GPT Version	Average Note + Prompt Length (Chars)	Input Cost (Per 1,000 Tokens)	Output Cost (Per 1,000 Tokens)	Expected Total Cost
BGR	GPT- 3.5	8583	$.0005	$.0015	$7.09
	GPT- 4 t	9933	$.01	$.03	$163.69
	GPT- 4o	9933	$.005	$.015	$81.85
	Calculated Total Cost of GPT- 3.5, GPT- 4 t, and GPT- 4o				*$252.63*
CP	GPT- 3.5	9676	$.0005	$.0015	$6.71
	GPT- 4 t	9676	$.01	$.03	$134.26
	GPT- 4o	9676	$.005	$.015	$67.13
	Calculated Total Cost of GPT- 3.5, GPT- 4 t, and GPT- 4o				*$208.10*
Calculated Total Cost of All Analyses on Both Datasets					$460.73

Calculated costs of all GPT extractions performed on both the BGR and CP datasets, broken down by GPT version

We calculated that the total cost of performing the extraction across all prompts was $252.63 for the BGR dataset and $208.10 for the CP cohort, leading to a total cost of $460.73. This cost encompassed utilization of 3 different GPT versions on two different datasets of > 3,000 clinical notes, demonstrating that clinical information extraction can be accurately performed for multiple iterations on thousands of notes at reasonable price point. However, recall that only a pre-specified subset of the participants EHR deemed most relevant to the topic area was used for this analysis.

## Discussion

Longitudinal assessment of functional ability is highly relevant to disease diagnosis and management, but the administration of standardized assessments can pose a substantial burden on patients, caregivers, and providers. For example, many of the reference standard measurements, such as the ADOS or Bayley, take too long to be administered in the clinic, and could potentially be shortened or replaced by LLM models applied to clinically acquired data. In this study, we created an AI pipeline to predict functional biomarkers, specifically verbal and ambulatory ability, and tested its performance in two independent cohorts, one comprising individuals with genetic causes of IDD (the BGR), and the other comprising individuals with CP. Our results demonstrate that an automated approach for predicting communication and ambulatory abilities from passively accrued EHR data, which is acquired during routine clinical care documentation, may provide a solution for extracting functional biomarkers for use in research studies and diverse clinical applications. For example, knowledge of the functional status of a patient is essential for tracking disease processes in single patients and groups of patients, as well as their response to therapy over time. Potential research uses for a rigorously evaluated and highly developed functional biomarker extraction pipeline include natural history studies for clinical trial readiness, particularly for rare diseases where patients may not all receive care at the same institution. Automated data extraction for functional outcomes may also yield particular benefits in resource-limited environments due to the passive nature of data collections.

When implementing our analysis pipeline on the BGR and CP cohorts, we used consistent criteria for note selection and analysis, such as including only notes of a sufficient length from specific author specialties, and excluding individuals with too few notes. We also provided the LLMs with the same prompts to elicit predictions on whether the individuals could “walk without aid” and “use words”. We found that for both cohorts the LLMs were highly successful at predicting the ability to “walk without aid”, but were more successful at predicting the ability to “use words” for the BGR cohort than the CP cohort. The consistent performance on the ambulatory ability prediction task across both cohorts is likely attributed to low ambiguity in the terminology used to describe ambulatory ability. Additionally, both cohorts utilize the GMFCS as a major component of the ground truth values, limiting divergence between prompt extracted ambulatory ability and our ground truth labels. Contrasting this, ground truth assessments of communication were different in the BGR and CP cohorts which made it difficult to use a single prompt to assess communication, leading to the varying performances across cohorts. The verbal ability prompt assessing use of words is more closely aligned to the ground truth communication assessment for the BGR cohort, as the CARS- 2 explicitly asks if the individual is using words (“Is the person you are rating using words?” and the telehealth-screener addresses the amount of words an individual uses (single words, sentences, etc.), which are both easily aligned to our question “does the individual use any words?” Contrasting this, the communication assessment in the CP cohort uses the VSS or CFCS. Of note, the VSS assesses the degree to which motor impairment affects an individual’s ability to produce oral speech and the CFCS describes an individual’s ability to both given and receive information to familiar and unfamiliar individuals. In this context, the ability to “use words” could be interpreted in multiple different ways including the ability to produce single words using oral motor speech or using a communication device or using signs. Future assessments of communication will likely require much more nuanced definitions of verbal ability that are customized to match the ground-truth assessments available for each clinical cohort.

Taken together, our results indicate that the LLM pipeline we developed is broadly generalizable, but that cohort-specific changes such as note inclusion criteria to maximize information content and minimize noise, and prompt engineering for clinical-relevance, may require performance optimization. Additionally, extraction methodology may need to be modified to account for cohort differences. For example, the CP cohort note template included identical note history sections for some patients receiving weekly physical therapy, which biased toward “no” responses to the ambulatory ability extraction question. This necessitated modifying the guidelines for mapping from note-level predictions to individual level predictions of ambulatory ability. Despite the need for considerations relating to cohort-specific changes, success of our LLM pipelines at extracting verbal and ambulatory ability from BGR clinical notes indicates that LLMs have the potential to build generalizable clinical extraction pipelines across multiple institutions with variable note-taking practices, and may therefore be hugely valuable for rare disease research.

In our LLM pipeline we mapped from predictions across various time points to a single individual-level prediction, which we recognized could be influenced by changes in patient functional abilities over time. To reduce the potential for this bias, we only included notes written when the individual was at least 3 years old (capturing individuals who have passed certain developmental stages), and we performed an analysis on notes written within 1.5 years of ground truth ascertainment across both cohorts. Despite these steps, we acknowledge that we may not have been able to control for all effects of individual development and/or improvement in ambulatory and verbal abilities.

One limitation of our study is the rapid evolution of LLMs. The LLMs we used were deployed behind the HIPAA-compliant firewalls of Washington University’s Azure tenant. While OpenAI does not commonly release training and detailed model information, GPT- 3.5 was released in November 2022 and is presumed to be the smallest in total parameter size and count of tokens in the training data compared to subsequent models. In March 2023, GPT- 4 was released and lauded for its notable increase in capability over GPT- 3.5. This first model released in the 4-series showed an ability to handle complex tasks and instructions with much improved performance [35–37]. Later, in November 2023, GPT- 4 T was released, and GPT- 4o was released in May 2024. Training of GPT- 4 T and GPT- 4o became more advanced and resulted in more capable models, both showing similar state of the art performances on several LLM text generation benchmarks, better than GPT- 4 [38].

We also note that incomplete representation for demographic groups may introduce unintentional bias to the pipelines and prompts, and impact the generalizability of the model to diverse populations. Although we included cohorts with representation across all demographic groups, the BGR included fewer individuals identifying as Black or African American (6.4%), Hispanic or Latine (6.4%), and as having mixed ancestry (2.4%) compared to the U.S. census population [39]. The CP cohort had better representation of female and Black or African American individuals, but the proportion of Hispanic or Latine identifying individuals was not recorded. If the pipelines in this study are to be utilized for clinical decision making, then additional investigation may be needed to ensure that there is no bias in the performance against underrepresented groups.

Cost analysis showed that the resources required to use GPT for automated biomarker identification are reasonable, albeit dependent on the chosen model and selective inclusion of notes. The cost of all LLM-based experiments is larger than that of alternative rule-based and machine learning methods, which may have little or no monetary cost; however, these other methods have a much higher time–cost associated. Future work will further examine the scalability of this work by exploring other open-source LLMs such as Llama, Mixtral, and FLAN that can be implemented with existing local computational resources, thus avoiding per-token costs as well as circumventing data privacy concerns.

A final limitation of our study was the potential bias of our two study cohorts, which were derived from academic institutions with likely higher incidences of functional deficits. Therefore, they may have more informative notes compared to IDD patients who have not had the same level of access to healthcare. Future plans include application of our pipeline to other populations, such as a general pediatric population (e.g. from primary care pediatricians), to determine if clinical notes from non-specialist care are sufficiently informative to predict functional ability. We also plan to determine if the model can predict complex information, such as the age at which individuals showed progress or decline in verbal and ambulatory ability, and more detailed and granular information such as the individual GMFCS levels rather than binary predictions. Application of these techniques show promise for informing the need for standardized data collection for natural history studies, as well as which assessments may best capture clinically meaningful change for clinical trials.

## Conclusions

In summary, here we demonstrate the successful design and application of LLM tools for extracting functional biomarker data from extant EHR data to make clinically meaningful predictions of verbal and ambulatory ability. We show that the questions ‘Can the individual walk without aid?’ and ‘Does the individual use any words?’ can be answered with good, though not perfect, fidelity from EHR data in two separate cohorts of patients with IDD or CP. We found that GPT- 4 t and GPT- 4o were superior to GPT- 3.5, with minor differences in performance between GPT- 4 t and GPT- 4o, and all can be accomplished at reasonable cost, which may be further reduced by translating these methods to open-source LLMs. The low-cost ability to extract functional biomarkers with LLM tools has extensive clinical and research applications, including generation of genotype–phenotype correlations, assessment of therapeutic interventions or harmful exposures, and identification of at-risk patients who may benefit from targeted treatments or therapies. Further development and optimization of LLM tools for extracting functional biomarkers offers an exciting opportunity to utilize the wealth of EHR data to efficiently advance research and clinical care for patients with IDDs as well as the population-at-large.

## Supplementary Information

Additonal file 1.

## Data Availability

The Brain Gene Registry dataset utilized in this article is available at https://braingeneregistry.wustl.edu/. The Cerebral Palsy dataset utilized is not publicly accessible.

## Abbreviations

IDDs: Intellectual and Developmental Disabilities
CP: Cerebral Palsy
LLMs: Large Language Models
BGR: Brain Gene Registry
EHR: Electronic Health Record
AI: Artificial Intelligence
NLP: Natural Language Processing
RNAP: Rapid Neurobehavioral Assessment Protocol
GPT: Generative Pre-trained Transformed
