# Supplementary material for: Automated extraction of functional biomarkers of verbal and ambulatory ability from multi-institutional clinical notes using large language models
Source: J Neurodev Disord. 2025 Apr 30;17:24. doi: 10.1186/s11689-025-09612-w (PMC12042395; doi:10.1186/s11689-025-09612-w)
Supplement: Supplementary file 1 — Additonal file 1. [file 11689_2025_9612_MOESM1_ESM.docx]

Supplementary Tables and Figures for the Manuscript: *Automated extraction of functional biomarkers of verbal and ambulatory ability from multi-institutional clinical notes using large language models*

**Supplementary Table 1: Clinician Identified Relevant Note Types.** In this table, the ‘Neuro Spec Qual Child Neurology’ specialty is an arbitrary specialty label given by a single institution for notes written by Child Neurologists.

|  | **Note Author Specialty** |
| --- | --- |
| **1** | Physical Therapy |
| **2** | Psychology |
| **3** | Occupational Therapy |
| **4** | Speech Therapy |
| **5** | Neuro Spec Qual Child Neurology |
| **6** | Psychiatry |
| **7** | Neurology |
| **8** | Psychiatry & Neurology |
| **9** | Child and Adolescent Psychiatry |
| **10** | Speech |
| **11** | Speech Language Pathology |
| **12** | Developmental Medicine |
| **13** | Developmental Pediatrics |
| **14** | Child Development |

**Supplementary Table 2**

|  |  | **All BGR Individuals with Profile** | **BGR Individual can walk without Aid?** | | **BGR Individual can use any words?** | | **Individuals in GPT Dataset** |
| --- | --- | --- | --- | --- | --- | --- | --- |
|  |  |  | **Yes** | **No** | **Yes** | **No** |  |
| **N** |  | 564 | 90 | 14 | 84 | 21 | 125 |
| **Age (sd)** |  | 11.4 (9.8) | 9.6 (3.9) | 8.2,(4.9) | 11.9 (6.3) | 11.4 (6.4) | 11.2 (6.1) |
| **Sex** | **Female** | 259 (45.9%) | 29 (32.2%) | 5 (35.7%) | 25 (29.8%) | 8 (38.1%) | 41 (32.8%) |
|  | **Male** | 305 (54.1%) | 61 (67.8%) | 9 (64.3%) | 59 (70.2%) | 13 (61.9%) | 84 (67.2%) |
|  | **Unknown/Not Reported** | 0 (0.0%) | 0 | 0 | 0 | 0 | 0 (0.00%) |
| **Ethnicity** | **Hispanic or Latine** | 56 (9.9%) | 4 (4.4%) | 3 (21.4%) | 4 (4.8%) | 0 | 8 (6.4%) |
|  | **Not Hispanic or Latine** | 406 (72.0%) | 67 (74.4%) | 10 (71.4%) | 67 (79.8%) | 14 (66.7%) | 94 (75.2%) |
|  | **Unknown/Not Reported** | 102 (18.1%) | 19 (21.1%) | 1 (7.1%) | 13 (15.5%) | 7 (33.3%) | 23 (18.4%) |
| **Race** | **Asian** | 25 (4.4%) | 4 (4.4%) | 1 (7.1%) | 4 (4.8%) | 0 | 5 (4.0%) |
|  | **Black or African American** | 27 (4.8%) | 7 (7.8%) | 1 (7.1%) | 3 (3.6%) | 2 (9.5%) | 8 (6.4%) |
|  | **More Than One Race** | 23 (4.1%) | 3 (3.3%) | 0 | 2 (2.4%) | 0 | 3 (2.4%) |
|  | **Other** | 19 (3.4%) | 1 (1.1%) | 0 | 1 (1.2%) | 0 | 1 (0.8%) |
|  | **Unknown/Not Reported** | 88 (15.6%) | 15 (16.7%) | 0 | 9 (10.7%) | 4 (19.1%) | 16 (12.8%) |
|  | **White** | 381 (67.6%) | 4 (4.4%) | 3 (21.4%) | 4 (4.8%) | 0 | 92 (73.6%) |

**Supplementary Table 3**

|  |  | **All CP Individuals with Profile** | **CP Individual can walk without Aid?** | | **Individuals in GPT Dataset** |
| --- | --- | --- | --- | --- | --- |
|  |  |  | **Yes** | **No** |  |
| **N** |  | 633 | 125 | 135 | 260 |
| **Age (sd)** |  | 8.5 (5.1) | 7.4 (5.1) | 8.0 (4.8) | 7.525 (4.91) |
| **Sex** | **Female** | 292 (46.1%) | 60 (48.0%) | 64 (47.4%) | 124 (47.7%) |
|  | **Male** | 337 (53.2%) | 64 (51.2%) | 71 (52.6%) | 135 (51.9%) |
|  | **Unknown Gender** | 4 (0.6%) | 1 (0.8%) | 0 (0.0%) | 1 (0.4%) |
| **Race** | **Asian** | 12 (1.9%) | 1 (0.8%) | 6 (4.4%) | 7 (2.7%) |
|  | **Black** | 113 (17.9%) | 31 (24.8%) | 31 (23.0%) | 62 (23.9%) |
|  | **Mixed** | 31 (4.9%) | 10 (8.0%) | 10 (7.4%) | 20 (7.7%) |
|  | **Other** | 12 (1.9%) | 3 (2.4%) | 2 (1.5%) | 5 (1.9%) |
|  | **Unknown Race** | 24 (3.8%) | 2 (1.6%) | 8 (5.9%) | 10 (3.9%) |
|  | **White** | 492 (77.7%) | 96 (76.8%) | 96 (71.1%) | 192 (73.9%) |

**Supplementary Table 4:** BGR Dataset GPT Extraction Performance with Macro-Avg F1 Scores

| Prompt and Note Set | Extraction Task | GPT 3.5 | | | GPT-4 | | | GPT-4o | | |
| --- | --- | --- | --- | --- | --- | --- | --- | --- | --- | --- |
|  |  | Avg Precision | Avg Recall | Macro Avg F1 | Avg Precision | Avg Recall | Macro Avg F1 | Avg Precision | Avg Recall | Macro Avg F1 |
| MCP-All | Ambulatory Ability | .60 | .69 | .59 | **.89** | **.91** | **.90** | .75 | .87 | .79 |
| BCP-All | Ambulatory Ability | .52 | .54 | .37 | **.67** | **.81** | **.68** | .63 | .76 | .61 |
| MCP-1.5Y | Ambulatory Ability | .63 | .74 | .64 | **.84** | **.86** | **.85** | .74 | .83 | .77 |
| MCP-All | Verbal Ability | .80 | .86 | .82 | .88 | .84 | .86 | **.87** | **.88** | **.87** |
| BCP-All | Verbal Ability | .78 | .83 | .80 | **.84** | **.89** | **.86** | .82 | .84 | .83 |
| MCP-1.5Y | Verbal Ability | .71 | .76 | .73 | .80 | .79 | .79 | **.82** | **.84** | **.83** |

**Supplementary Table 5:** CP Dataset GPT Extraction Performance with Macro-Avg F1 Scores

| Prompt and Note Set | Extraction Task | GPT 3.5 | | | GPT-4 | | | GPT-4o | | |
| --- | --- | --- | --- | --- | --- | --- | --- | --- | --- | --- |
|  |  | Avg Precision | Avg Recall | Macro Avg F1 | Avg Precision | Avg Recall | Macro Avg F1 | Avg Precision | Avg Recall | Macro Avg F1 |
| MCP-CP-VSS | Verbal Ability | .69 | .63 | .53 | **.72** | .71 | .66 | **.74** | **.73** | **.68** |
| MCP-CP-CFCS | Verbal Ability | .69 | .63 | .54 | .68 | .67 | .63 | **.72** | **.71** | **.66** |
| MCP-CP-GMFCS | Ambulatory Ability | .73 | .75 | .69 | .86 | 0.9 | .87 | **.88** | **.91** | **.89** |

**Supplementary Figure 1: Utilizing Diagnoses Codes to Determine Patient Verbal and Ambulatory Ability.** Expert physicians identified ICD codes present within the BGR dataset that suggested a lack of ability to use any words or walk without aid. Five ICD-10 codes (R26.2, Z99.3, Z74.09, R26.9, R27.0) relating to ambulatory ability were identified, and presence of a single code mapped to ‘No ability to walk without aid’. Similarly, five ICD codes (R47.01, R47.89, F81.89, F80.1, F80.0) were identified that suggested some lack of ability to use any words, and presence of a single code led to a ‘No’ response to the verbal ability question. This evaluation was performed for BGR individuals with ground truth values and diagnoses codes available. Overall, mapped ICD codes led to poor performance in determining verbal and ambulatory abilities compared to the ‘ground truth’ as determined by RNAP, highlighting an underutilization of codes that address verbal and ambulatory abilities.

**
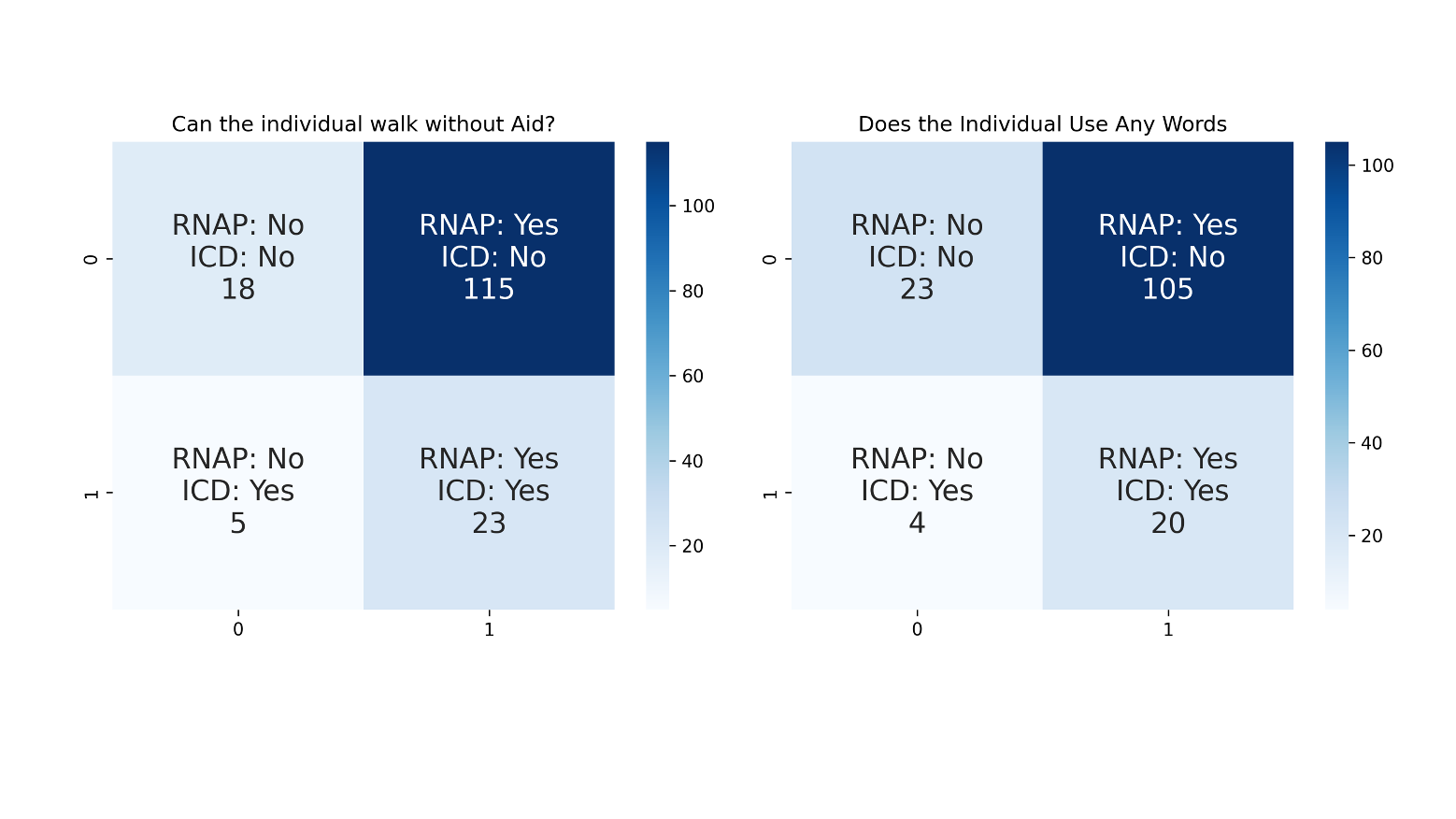
**
